# Outcomes of acute kidney injury in patients receiving extracorporeal membrane oxygenation during the COVID-19 pandemic: a prospective, observational, and multi-center study

**DOI:** 10.1080/0886022X.2025.2570817

**Published:** 2025-10-20

**Authors:** Ali AlSahow, Omar Alkandari, Heba AlRajab, Bassam AlHelal, Anas AlYousef, Ahmed AlQallaf, Yousif Bahbahani, Abdulrahman AlKandari, Gamal Nessim, Bassem Mashal, Ahmad Mazroue, Mohamed ElAbbadi, Alaa Abdelmoteleb, Ali Abdelzaher, Mohamed Abdellatif, Ziad ElHusseini, Ahmed Abdelrady, Emad Abdalla

**Affiliations:** a Division of Nephrology, Jahra Hospital, Al Jahra, Kuwait; b Division of Pediatric Nephrology, Mubarak Hospital, Jabriya, Kuwait; c Division of Nephrology, Farwaniya Hospital, Sabah Al Nasser, Kuwait; d Division of Nephrology, Adan Hospital, Hadiya, Kuwait; e Division of Nephrology, Amiri Hospital, Kuwait City, Kuwait; f Division of Nephrology, Jaber Hospital, Kuwait City, Kuwait; g Division of Nephrology, Mubarak Hospital, Jabriya, Kuwait; h Division of Nephrology, Chest Diseases Hospital, Shuwaikh Industrial, Kuwait

**Keywords:** Acute kidney injury, extracorporeal membrane oxygenation, mortality, dialysis, Kuwait

## Abstract

**Introduction:**

Extracorporeal membrane oxygenation (ECMO) is a life-saving therapy in severe respiratory and/or cardiovascular failure. Acute kidney injury (AKI) is a frequent complication of ECMO that increases morbidity and mortality. We report the outcomes of patients with AKI who received ECMO.

**Methods:**

Clinical, management, and 30-d kidney and patient outcome data of adult inpatients with AKI who received ECMO in seven public hospitals in Kuwait from 1 January to 31 December 2021, were prospectively collected and analyzed.

**Results:**

There were 3,744 AKI referrals to nephrology during study period, of which 121 received ECMO (3.2%). Patients with AKI on ECMO had a mean age of 56.3 years and a mean baseline eGFR of 81.6 mL/min. Preexisting chronic kidney disease was reported in 21.5% of patients, diabetes in 58.7%, and hypertension in 48%. COVID-19 infection contributed to AKI in 69% of the cases. AKI developed before ECMO initiation in 62% of cases. ECMO was veno-venous in 90% of cases. Dialysis was performed in 92% of cases, 97% of which was continuous modality. Mechanical ventilation was required in 94.2% of patients (all on inotropic support). At 30 d, 86.8% of the cohort died (91% of the deceased were on dialysis), 5% remained on dialysis, and only 3.3% recovered kidney function completely.

**Conclusions:**

AKI in patients receiving ECMO was associated with a high need for dialysis, and a high mortality rate. COVID-19 pandemic may have contributed to this outcome. ECMO modality, and whether AKI was pre or post ECMO did not affect the outcome.

## Introduction

Extracorporeal membrane oxygenation (ECMO) whether veno-arterial (VA-ECMO) or veno-venous (VV-ECMO), is a life-saving therapy for patients with severe respiratory and/or cardiovascular failure [1]. However, the incidence of severe acute kidney injury (AKI) among ECMO patients is high regardless of whether dialysis is required, which significantly increases morbidity and mortality. AKI is reportedly more common with VA-ECMO than with VV-ECMO. Furthermore, AKI is most often present before ECMO initiation [1–3]. In this article, we report the rate of AKI in patients receiving ECMO and the rate of ECMO utilization in patients with AKI in public hospitals in Kuwait. We also report patient and kidney outcomes in patients who developed AKI and received ECMO and compare them to a matching group of patients with AKI who did not require ECMO.

## Methods

This prospective observational study was conducted in seven Ministry of Health (MoH) hospitals in Kuwait from 1 January 2021 to 31 December 2021 after obtaining ethical approval. First, all AKI cases referred to nephrology during the study period were recorded. Patients above the age of 18 years, with native kidneys and an estimated glomerular filtration rate (eGFR) above 10 mL/min were included. Kidney transplant recipients, dialysis patients, and chronic kidney disease (CKD) patients not on dialysis but with an eGFR less than 10 mL/min (or even higher if they had preemptive dialysis access) were excluded. Patients with pre-renal AKI (recovery in less than 24 h) and those who died within 24 h of consultation were also excluded. The total number of cases was 3,744. All cases of AKI that received ECMO were then extracted for further analysis. Patient demographics, clinical profiles, and dialytic and non-dialytic management data were collected, and patients were followed up for 30 d, even after discharge (or for less than 30 d if recovery or death occurred earlier), to record kidney and patient outcomes. The Kidney Disease: Improving Global Outcome (KDIGO) definition for AKI [4] and the KDIGO definition for CKD [5] for patients with preexisting CKD were used. Lack of kidney recovery was defined as no change in eGFR from the nadir at 30 d, and complete recovery was defined as the return of eGFR to baseline within 5 mL/min.

For statistical analysis, continuous variables were assessed for distribution and expressed as mean ± standard deviation (SD) or median and interquartile range (IQR) as appropriate. Categorical variables were expressed as numbers and percentages (%). A *p* value of < 0.05 was considered statistically significant. The differences between groups were determined by student *t*-test, Wilcoxon rank-sum test, the chi-squared test, or Fisher’s exact test, as appropriate. We used a propensity score matching to adjust for possible confounding bias. The following variables were used for the propensity score matching: age, sex, nationality (Kuwaiti *vs.* non-Kuwaiti), baseline eGFR, baseline Hb, COVID-19 and history of diabetes, hypertension or cardiovascular disease. We used a 1:1 ratio, nearest-neighbor matching (with a caliper of 0.2 on the PS to eliminate bias) with no replacement. We compared the two groups by the standardized differences, where 0.2, 0.5, and 0.8 considered as small, medium and large differences, respectively. We used *p* value of <0.05 as a statistical significance. STATA statistical software version 17 (Stata Corp LLC., College Station, TX) was used for statistical analysis. STROBE guidelines for cohort studies were followed.

## Results

**Figure 1.**
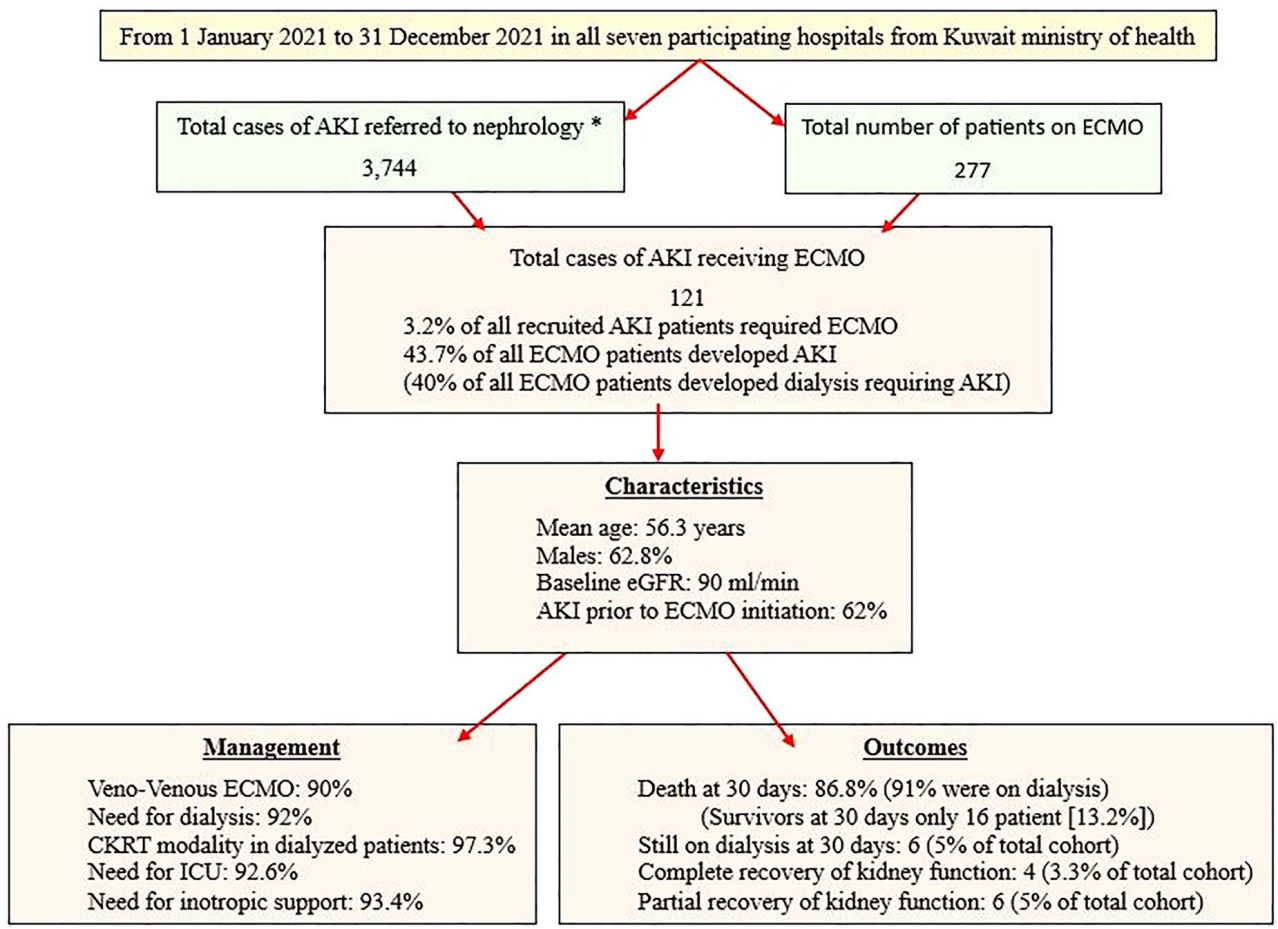
Flow chart showing the method of selecting our cohort, their numbers and percentages, as well as a summary of their management and outcomes. AKI: acute kidney injury; ECMO: extracorporeal membrane oxygenation; CKRT: continuous kidney replacement therapy; ICU: intensive care unit. *Please see the inclusion and exclusion criteria in the Methods section.

The total number of AKI referrals to the nephrology service in all participating hospitals was 3,744, and the total number of patients who received ECMO in the same hospitals was 277 during the study period. However, the number of patients who had AKI and received ECMO was 121. This represents 3.2% of all AKI cases (121 out of 3,744) and 43.7% of ECMO cases (121 out of 277). AKI developed before ECMO initiation in 62% of the patients in this group. Figure 1 provides further details.

Table 1 describes the basic characteristics of patients who had AKI and were on ECMO. The mean age of the ECMO group was 56.3 years with only 26.5% of the cohort being older than 65 years. The mean baseline eGFR was 81.6 mL/min/1.73 m^2^, with preexisting CKD reported in only 21.5% of the patients, mainly due to diabetic kidney disease. Men accounted for 62.8% of the cohort, and 91.7% of the AKI cases were hospital-acquired. Diabetes was reported in 58.7% and hypertension reported in 48%, and 28% of the cohort had no comorbidities. Acute tubular necrosis and COVID-19 were the most common presumed causes of AKI, accounting for 76.0%, and 69.4% of cases, respectively. Sepsis and renal hypoperfusion were the most common precipitating factors for AKI, reported in 89.3%, and 45.5% of cases, respectively.

**Table 1.** Basic characteristics of patients with acute kidney injury on ECMO and their non-ECMO matched cohort.

Variable	Not on ECMO (*n* = 121)	On ECMO (*n* = 121)	Standard difference (*p* value)
Age, year, mean ± SD	56.5 ± 15.6	56.3 ± 15.6	−0.014 (0.9)
Male sex, *n* (%)	77 (63.6)	76 (62.8)	−0.017 (0.9)
Kuwaiti nationality, *n* (%)	66 (54.6)	63 (52.1)	−0.05 (0.7)
Baseline eGFR, mL/min/1.73 m^2^, median [IQR]	89 [63–95]	90 [65–98]	0.099 (0.24)
Baseline Hb, g/L, mean ± SD	103.0 ± 20.9	99.7 ± 19.0	−0.16 (0.2)
Comorbidities*			
Age > 65, *n* (%)	40 (33.1)	32 (26.5)	−0.14 (0.26)
Diabetes, *n* (%)	71 (58.7)	71 (58.7)	<0.001 (1.0)
Hypertension, *n* (%)	54 (44.6)	58 (47.9)	0.066 (0.6)
CVD, *n* (%)^#^	38 (31.4)	39 (32.2)	0.018 (0.9)
No comorbidities, *n* (%)	34 (28.1)	34 (28.1)	<0.001 (1.0)
Cause of AKI*			
Ischemic/toxic ATN, *n* (%)	89 (73.6)	92 (76.0)	0.057 (0.66)
COVID-19 related, *n* (%)	87 (71.9)	84 (69.4)	−0.054 (0.67)
Drug-related injury, *n* (%)	24 (19.8)	7 (5.8)	−0.428 (0.001)
Precipitating factors*			
Sepsis, *n* (%)	98 (81.0)	108 (89.3)	0.233 (0.07)
Volume depletion, *n* (%)	68 (56.2)	55 (45.5)	−0.215 (0.1)
Drug toxicity, *n* (%)	32 (26.5)	6 (5.0)	−0.62 (<0.001)

The non-ECMO AKI group was matched for age, sex, nationality, baseline eGFR, baseline hemoglobin, COVID-19, and presence of diabetes, hypertension, and cardiovascular disease.

Values are given as mean ± SD for continuous variables and as number (percentage) for categorical variables.

*More than 1 answer per patient was permissible, that explains why total % is greater than 100%.

ECMO: extracorporeal membrane oxygenation; AKI: acute kidney injury; eGFR: estimated glomerular filtration rate; ATN: acute tubular necrosis

^#^Cardiovascular disease: Coronary artery disease, heart failure, and peripheral arterial disease.

Table 2 details the non-dialytic and dialytic management of the ECMO group. The vast majority of the cohort (92.6%) was in the intensive care unit (ICU), 94.2% of patients were mechanically ventilated, and 93% of patients required intravenous (IV) inotropic support. IV fluids were used in 64.5% of patients prior to dialysis initiation. ECMO was VV in 90% of cases. Hemodialysis (HD) was provided for 91.7% of cases, with continuous kidney replacement therapy (CKRT) as the modality of choice in almost all cases (97.3%). Vascular access for dialysis was right internal jugular in 53% and femoral in 40.5% of cases. In comparison, the matching non-ECMO AKI cohort had lower needs for inotropic support, lower need for mechanical ventilation, and lower need for dialysis.

**Table 2.** Non-dialytic and dialytic management of patients with acute kidney injury on ECMO and their non-ECMO matched cohort.

Variable	Not on ECMO (*n* = 121)	On ECMO (*n* = 121)	Standard difference (*p* value)
IV Fluids*			
None, *n* (%)	24 (19.8)	43 (35.5)	−0.355 (0.006)
Normal saline, *n* (%)	78 (64.5)	63 (52.1)	−0.252 (0.051)
Bicarbonate/RL/Plasmalyte, *n* (%)	28 (23.1)	12 (9.9)	−0.36 (0.006)
Blood/blood products, *n* (%)	14 (11.6)	25 (20.7)	0.248 (0.054)
Any IV fluid or combination, *n* (%)	97 (80.2)	78 (64.5)	−0.355 (0.006)
IV Diuretics*			
None, *n* (%)	43 (35.5)	42 (34.7)	
IV diuretics (mostly loop diuretics), *n* (%)	78 (64.5)	79 (65,3)	0.017 (0.9)
IV Vasopressors*			
None, *n* (%)	42 (34.7)	8 (6.6)	0.737 (<0.001)
Noradrenaline, *n* (%)	72 (59.5)	98 (81.0)	0.482 (<0.001)
Dopamine, *n* (%)	13 (10.7)	40 (33.1)	0.558 (<0.001)
Vasopressin, *n* (%)	39 (32.2)	57 (47.1)	0.306 (0.02)
Dobutamine, *n* (%)	2 (1.7)	11 (9.1)	0.333 (0.1)
Any IV vasopressor or combination, *n* (%)	79 (65.3)	113 (93.4)	0.737 (<0.001)
Dialysis Indication	60 (49.6%)	111 (91.7%)	1.04 (<0.001)
Volume overload, *n* (%)	48 (80.0%)	90 (81.1%)	0.027 (0.86)
Electrolytes/acid-base disorders, *n* (%)	53 (88.3%)	97 (87.4%)	−0.029 (0.85)
Initial Modality			
Intermittent HD, *n* (%)	4 (6.7%)	2 (1.8%)	−0.242 (0.1)
CKRT, *n* (%)	56 (93.3%)	108 (97.3%)	0.187 (0.2)
Other management			
Steroid use, *n* (%)	82 (67.8)	84 (69.4)	0.035 (0.8)
Admission to ICU, *n* (%)	82 (67.8)	112 (92.6)	0.651 (<0.001)
Mechanical ventilation, *n* (%)	81 (66.9)	114 (94.2)	0.731 (<0.001)

The non-ECMO AKI group was matched for age, sex, nationality, baseline eGFR, baseline hemoglobin, COVID-19, and presence of diabetes, hypertension, and cardiovascular disease.

*More than 1 answer per patient was possible, that is why total % is greater than 100%.

ECMO: extracorporeal membrane oxygenation; AKI: acute kidney injury; ECMO: Extracorporeal Membrane Oxygenation; IV: intravenous; ICU: Intensive Care Unit; CKRT: Continuous Kidney Replacement Therapy; HD: hemodialysis.

Table 3 shows patient and kidney outcomes at 30 d. Of the cohort, 86.8% died (91% of those who died were on dialysis), with sepsis as the most common cause of death (81.2%), compared to 49.6% death rate in the matching non-ECMO AKI group. Of the 16 patients who remained alive at 30 d, six patients (5%) remained on dialysis, and only four patients (3.3%) recovered kidney function completely (3/4 had AKI post ECMO). Patients who were alive and off dialysis at 30 d (8.3%) had a median eGFR of 65 mL/min. Comparative analysis showed that in the ECMO group, the mortality rate was 86.5% in the COVID-19 positive patients *vs.* 86.9% in the non-COVID-19 patients (*p* = 0.950), whereas in the non-ECMO group, the mortality rate was 58.6% in the COVID-19 positive patients *vs*. 26.5% in the non-COVID-19 patients (*p* = 0.001), suggesting that COVID-19 did not significantly influence the mortality rate in patients receiving ECMO.

**Table 3.** Kidney and patient outcome of acute kidney injury at 30 d for patients on ECMO and their non-ECMO matched cohort.

Variable	Not on ECMO (*n* = 121)	ECMO (*n* = 121)	Standard difference (*p* value)
Cause of death*	60 (49.6%)	105 (86.8%)	0.867 (<0.001)
Infection/sepsis, *n* (%)	51 (42.2)	85 (81.2%)	0.588 (<0.001)
Cardiovascular, *n* (%)	6 (5.0)	20 (19.0%)	0.379 (0.006)
Dialysis status at 30 d	60 (49.6%)	111 (91.7%)	
Still on dialysis, *n* (%)	12 (20.0%)	20 (18.0%)	−0.05 (0.45)
Dialysis not further needed, *n* (%)	11 (18.3%)	6 (5.4%)	−0.405 (0.009)
Died while on dialysis, *n* (%)	37 (61.7%)	85 (76.6%)	0.325 (0.03)
Kidney function at 30 d**			
No recovery, *n* (%)	20 (32.8%)	6 (37.5%)	0.12 (0.47)
Partial recovery, *n* (%)	19 (31.1%)	6 (37.5%)	0-.12 (0.42)
Complete recovery, *n* (%)	22 (36.1%)	4 (25.0%)	−0.25 (0.3)
Median (IQR) eGFR at 30 d***	66 (23–91)	65 (48–87)	0.294 (0.4)

The non-ECMO AKI group was matched for age, sex, nationality, baseline eGFR, baseline hemoglobin, COVID-19, and presence of diabetes, hypertension, and cardiovascular disease.

Values are given as mean ± SD for continuous variables and as number (percentage) for categorical variables.

*More than 1 answer per patient was possible, that is why total % is greater than 100%.

**N = 16; after exclusion of deceased patients.

***N = 10; after exclusion of deceased patients and patients still on dialysis at 30 d.

eGFR: estimated glomerular filtration rate

## Discussion

ECMO, in its two main configurations (VA-ECMO and VV-ECMO), is a life-saving therapy for patients with severe respiratory and/or cardiovascular failure, and its use has increased substantially over the past decade [1]. However, it is frequently complicated by AKI, which increases morbidity and mortality. In this study, we showed that only 3.2% of all AKI cases received ECMO during the study period. We could not find a study that reported the percentage of AKI patients who required ECMO for comparison. The reported incidence of AKI in patients treated with ECMO ranges from 26% to 85% owing to differences in patient characteristics, AKI definition, and clinical settings. The pooled estimated incidence rates of AKI according to KDIGO criteria and severe AKI requiring KRT were 68.2% and 44.9%, respectively [1,2]. We showed that in patients who received ECMO, the incidence of AKI was 43.7% and the incidence of dialysis-requiring AKI was 40% during the study period. Accordingly, we observed a lower rate of AKI in patients receiving ECMO than what is reported in the literature but a similar rate of AKI requiring dialysis in patients receiving ECMO [6,7]. The mechanism of AKI in patients receiving ECMO is multifactorial. Prior to ECMO initiation, hemodynamic instability, nephrotoxins, and critical illness-related factors, such as hypoxemia, bleeding, ischemia, infection, and coagulopathy, can all cause AKI. In addition, heart failure factors, such as low cardiac output, high intra-abdominal pressure, and renal congestion, may contribute to AKI [1–3,8]. Post ECMO initiation, AKI can induce systemic inflammation and neurohormonal dysregulation by releasing immune-mediated factors through exposure to membranes and due to nonpulsatile renal blood flow, blood shear stress, air and blood embolisms, and hemolysis [1,9,10]. The literature that directly addresses the risk factors for AKI in the context of ECMO is limited. However, the risk factors for AKI in critically ill patients reported in the literature that may be relevant to ECMO may include male sex, older age, and higher comorbidity burden [2,3,8,11]. Our study showed more male involvement than female involvement and a higher comorbidity burden. However, the impact of patient age is controversial. Old age is a known risk factor for AKI in ECMO [2,8,11], but one study reported that age was neither a risk factor for AKI nor did it modify the effect of AKI on mortality in ECMO patients [8]. We must point out that the mean age of our patients, which was 56 years, was higher than the mean age of patients recruited for that study, which was 51 years.

Due to the severity of the underlying disease in patients receiving ECMO, hospital mortality is frequently high and substantially higher than for other etiologies of AKI in ICUs [10,12]. Most studies have demonstrated poor survival rates for patients with AKI who are offered ECMO [11,12]. The reported overall in-hospital ECMO-associated mortality was 57.1%, higher in those with AKI (63.7%), and even higher in AKI patients who required dialysis [2,3,8]. A retrospective analysis from Germany showed a 3-month mortality rate of 83% for ECMO patients with AKI requiring dialysis [13]. We reported an even higher mortality rate (∼87% at 30 d) in patients with AKI who were treated with ECMO. This could be partly explained by the higher comorbidity burden and higher mean age of our cohort compared to those in many published studies. However, another important contributing factor to the high mortality rate in our cohort is that COVID-19 was a cause of AKI in almost 70% of our cases. Almost half of the patients hospitalized for COVID-19 experience AKI, similar to high-risk AKI settings, such as cardiac surgery or sepsis, and AKI is strongly linked to mortality in COVID-19 [14]. The mortality rate of COVID-19 patients receiving ECMO is as high as 83.3% [15]. The need for KRT increased the mortality rates of COVID-19 patients with AKI in the ICU receiving ECMO [16]. Relevant clinical evidence on the use of ECMO in COVID-19 patients is scarce, and the timing, indications, management, benefits, and risks of ECMO are still controversial. The long-term renal prognosis of survivors is uncertain [17]. Although some survivors may be liberated from dialysis at hospital discharge [18], less than half of AKI patients requiring dialysis who were on ECMO and survived experienced complete recovery of kidney function [19]. Moreover, the 90-d mortality rate remains high for AKI patients who received ECMO [3]. In our cohort at 30 d, only nine of 111 (8.1%) patients who received dialysis were still on dialysis, and only four of 16 survivors (25%) recovered kidney function completely.

AKI is most often reported to develop before ECMO initiation, and it developed pre-ECMO in 62% of our cohort. Although AKI that develops after ECMO initiation was thought to be associated with worse outcomes, more recent studies showed no difference in mortality between AKI pre- and post-ECMO initiation [5]. This agrees with our finding of a lack of a significant difference in mortality between pre-ECMO AKI and post-ECMO AKI. AKI is also reported to be more common with VA-ECMO than with VV-ECMO (61% *vs*. 46%) [1]. One of the main differences between the two modalities is that VA-ECMO provides a smaller pulse pressure, while VV-ECMO maintains pulsatile cardiac output. Pulsatile flow may protect the microcirculation and kidney perfusion [12]. In our cohort, ECMO was VV in 90% of cases and had no significant benefit for survival, but the observational nature limits the generalizability of the outcome.

## Conclusion

The percentage of patients with AKI who may require ECMO is small; however, the percentage of patients on ECMO complicated by AKI is high. AKI in patients receiving ECMO is associated with a very high need for ICU admission, mechanical ventilation, and dialysis and a very high mortality rate. The reasonable baseline eGFR for patients, dominance of the VV-ECMO modality, and the fact that most AKI cases were pre-ECMO initiation had no impact on this dismal outcome. Our findings suggest that individualization of ECMO initiation in patients with dialysis-requiring AKI, whether AKI developed prior to or after ECMO initiation, especially in patients above the age of 56, is advised. The coexistence of COVID-19 and need for ECMO in patients with AKI significantly worsened the 30-d patients’ outcomes.

## Data Availability

Deidentified participant data, as well as the study protocol, can be provided by the corresponding author after approval of a reasonable request, with the publication of this article.

## Limitations of the study

The study was limited by the small number of cases and the lack of a control group, such as a similar group of AKI patients without the need for ECMO, or vice versa (cases of ECMO without AKI). However, since the mortality associated with ECMO in patients with AKI was extremely high, including a control group might not have made a significant difference.
